# G-Protein-Coupled Receptor (GPCR) Signaling in the Carotid Body: Roles in Hypoxia and Cardiovascular and Respiratory Disease

**DOI:** 10.3390/ijms21176012

**Published:** 2020-08-20

**Authors:** Hayyaf S. Aldossary, Abdulaziz A. Alzahrani, Demitris Nathanael, Eyas A. Alhuthail, Clare J. Ray, Nikolaos Batis, Prem Kumar, Andrew M. Coney, Andrew P. Holmes

**Affiliations:** 1 Institute of Clinical Sciences, University of Birmingham, Birmingham B15 2TT, UK; HXA807@student.bham.ac.uk (H.S.A.); AAA717@student.bham.ac.uk (A.A.A.); demitris.nathanael@gmail.com (D.N.); EXA833@student.bham.ac.uk (E.A.A.); c.j.ray@bham.ac.uk (C.J.R.); p.kumar@bham.ac.uk (P.K.); a.m.coney@bham.ac.uk (A.M.C.); 2 College of Medicine, Basic Medical Sciences, King Saud bin Abdulaziz University for Health Sciences, Riyadh 11481, Saudi Arabia; 3 Respiratory Care Department, Faculty of Applied Medical Sciences, Umm Al-Qura University, Makkah 24381, Saudi Arabia; 4 Collage of Sciences and Health Professions, Basic Sciences Department, King Saud bin Abdulaziz University for Health Sciences, Riyadh 11481, Saudi Arabia; 5 Institute of Cancer and Genomic Sciences, University of Birmingham, Birmingham B15 2TT, UK; N.Batis@bham.ac.uk; 6 Institute of Cardiovascular Sciences, University of Birmingham, Birmingham B15 2TT, UK

**Keywords:** G-protein, GPCR, carotid body, hypoxia, hypertension, drug-discovery

## Abstract

The carotid body (CB) is an important organ located at the carotid bifurcation that constantly monitors the blood supplying the brain. During hypoxia, the CB immediately triggers an alarm in the form of nerve impulses sent to the brain. This activates protective reflexes including hyperventilation, tachycardia and vasoconstriction, to ensure blood and oxygen delivery to the brain and vital organs. However, in certain conditions, including obstructive sleep apnea, heart failure and essential/spontaneous hypertension, the CB becomes hyperactive, promoting neurogenic hypertension and arrhythmia. G-protein-coupled receptors (GPCRs) are very highly expressed in the CB and have key roles in mediating baseline CB activity and hypoxic sensitivity. Here, we provide a brief overview of the numerous GPCRs that are expressed in the CB, their mechanism of action and downstream effects. Furthermore, we will address how these GPCRs and signaling pathways may contribute to CB hyperactivity and cardiovascular and respiratory disease. GPCRs are a major target for drug discovery development. This information highlights specific GPCRs that could be targeted by novel or existing drugs to enable more personalized treatment of CB-mediated cardiovascular and respiratory disease.

## 1. Introduction

The carotid body (CB) is a vital sensory organ, located near the carotid bifurcation, that constantly monitors blood supplying the brain [1]. The CB is stimulated by acute hypoxia, upon which it rapidly activates vital cardiovascular and respiratory reflexes, including peripheral vasoconstriction, elevated heart rate and increased breathing [2]. These ensure that sufficient blood-oxygen is delivered to the brain to support survival. It is now apparent that there are numerous G-protein-coupled receptors (GPCRs) expressed in the CB, and that modulation of these receptors is able to alter baseline CB activity and the sensitivity to hypoxia. Indeed, components of GPCR signaling pathways represent some of the most highly expressed genes in the rodent CB [3]. Three different types of GPCR alpha subunit have been identified to date in the CB: G_αs_, G_αi_ and G_αq_, and each one of these subtypes has a unique mechanism of action. Activation of G_αs_ will lead to transmembrane adenylyl cyclase (tmAC) stimulation, which in turn leads to an increase in intracellular cyclic adenosine monophosphate (cAMP). In contrast, the activation of G_αi_ will lead to the inhibition of tmAC activity, which in turn decreases cAMP [4,5]. The activation of G_αq_ stimulates phospholipase C (PLC), leading to the production of diacylglycerol (DAG) and inositol trisphosphate (IP_3_), which can modify [Ca^2+^]_i_, protein kinase activity, ion channel function and potentially also reactive oxygen species (ROS) generation [1]. Importantly, the CB has gained attention clinically due to its hyperactivity in a number of conditions, including obstructive sleep apnea (OSA), heart failure (HF) and essential/spontaneous hypertension (SH), in which it promotes neurogenic hypertension and arrhythmia. Multiple GPCRs have been implicated in CB hyperactivity, and these may offer key targets for novel drug discovery or repurposing. However, as yet, there are no drugs used clinically that directly target the CB, and this will be required in order to provide better and more personalized treatment of CB-mediated cardiovascular and respiratory disease. The aim of this review is to briefly explore the role of the major GPCRs and associated ligands in the CB, both in mediating normal CB function, hypoxic sensitivity and CB hyperactivity.

## 2. G_αs_ and G_αi_ Protein-Coupled Receptor Signaling in the Carotid Body

### 2.1. Adenosine and CD73

Adenosine is a precursor and a breakdown product of an important excitatory neurotransmitter in the CB, ATP, which is tonically released from the type I cell [6]. Extracellular concentrations of adenosine and ATP are reported to be approximately 20 pmol/CB and 4 pmol/CB respectively, in normoxia and increase by 174% and 147% in mild hypoxia in the rat CB [7,8]. A_2A_ and A_2B_-receptor mRNA has been isolated from rat CBs, and immunocytochemical and in situ hybridization techniques have shown that A_2A_- and A_2B_-receptors are present on the type I cell and A_2A_-receptors are expressed on post-synaptic sensory fibers [9,10,11,12]. In contrast, A_1_- and A_3_-receptors do not appear to be present in the CB [9,12].

CB activation by exogenous adenosine in vivo evokes an acute increase in respiratory frequency, tidal volume and minute ventilation in the rat [13]. More recent studies have now also confirmed that acute adenosine administration elevates ventilation in humans [14]. Exogenous adenosine is capable of activating all aspects of the CB chemotransduction cascade, including inhibition of Twik-related acid-sensitive K^+^ (TASK) and voltage-gated K^+^ channels, elevation of [Ca^2+^]_i_, promotion of neurotransmitter release and stimulation of chemoafferent fibers [11,15,16,17].

Endogenous adenosine plays an important role in establishing baseline neurotransmitter release and chemoafferent activity in normoxia [11,18]. The non-selective A_2_-receptor antagonist 8-(p-Sulfophenyl)theophylline (8-SPT) causes more than 90% depletion in chemoafferent frequency when measured in the ex vivo CB preparation [18]. This is particularly important given that it is the rise in baseline chemoafferent activity that most likely promotes chronic reflex stimulation and neurogenic hypertension associated with OSA, SH and HF. Initial reports suggest that caffeine (an adenosine receptor antagonist), does not modify the basal chemoafferent frequency in animals following exposure to chronic intermittent hypoxia (CIH), a robust model of OSA [19]. However, it must be noted that these animals displayed a reduction rather than elevation in baseline nerve activity, which is not characteristic of other studies using CIH. More experiments are definitely warranted to evaluate this further as well as a potential role for adenosine in mediating CB hyperactivity in SH and HF.

Adenosine also has an important role in mediating CB responses to hypoxia [20,21,22] and hypercapnia [18,23]. Inhibition of adenosine receptors has a greater impact at low-intensity levels of hypoxia [8], suggesting that adenosine is important in establishing CB hypoxic sensitivity and/or that adenosine release predominates under milder hypoxic conditions. However, the exact signaling mechanism remains controversial. Both A_2A_- and A_2B_-receptors are coupled by the G_αs_ protein, thus a role for adenylyl cyclase (AC) activation and cAMP accumulation appear highly likely [5,24]. In the whole CB preparation, A_2B_-, but not A_2A_-receptor antagonists depress catecholamine secretion in hypoxia [11,25]. In these same studies, it was reported that both A_2A_- and A_2B_-receptor antagonists reduce chemoafferent frequency in hypoxia, leading the authors to conclude that adenosine acts on pre-synaptic A_2B_- and post synaptic A_2A_-receptors. This is supported by the finding that A_2B_- not A_2A_-receptor inhibition suppresses type I cell catecholamine secretion and rises in [Ca^2+^]_i_ induced by adenosine in co-cultures with petrosal neurons [10]. However, in an earlier study, it was observed that A_2A_-receptor antagonism almost completely abolished the adenosine-mediated elevation in Ca^2+^, an effect that was mimicked by the protein kinase A (PKA) inhibitor H89 [17]. Other studies have shown that H89 and other PKA inhibitors do not significantly alter the catecholamine release caused by hypoxia [26]. This has led to the hypothesis that whilst increased adenosine and cAMP are important, they may be acting independently of PKA. Alternative messengers of cAMP include exchange proteins activated by cAMP (EPACs), which when activated, can overcome the inhibition of AC in the CB [26]. EPAC is suggested to be a key regulator of exocytotic machinery and K^+^ channels. Although no direct link between adenosine and EPAC has yet been established, as studies so far have only looked at the link between hypoxia and EPAC, a link is plausible and could be the focus of future investigations. Another target could be the hyperpolarization-activated cation current I_h_ reported to be activated via an A_2A_-receptor and cAMP-dependent mechanism on the post-synaptic site [27].

An important consideration is the actual source of adenosine in normoxia and hypoxia. It has been suggested that adenosine may be formed inside type I cells by the breakdown of cAMP and ATP and subsequently released through the bidirectional equilibrative nucleoside transporter (ENT) into the synapse [7,8]. This source of adenosine might be expected to increase proportionally during hypoxia as oxidative phosphorylation decreases and cAMP increases. It is therefore somewhat surprising that the functional impact of adenosine is more apparent at low- rather than high-intensity hypoxia [8]. Furthermore, our own experiments found that pharmacological inhibition of the ENT did not modify chemoafferent frequency in either normoxia or hypoxia [22]. This is possibly suggestive of an alternative source of adenosine.

**Figure 1:**
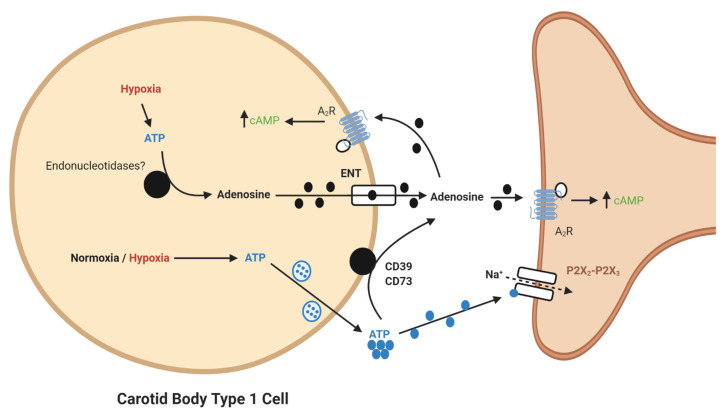
Schematic illustration of ecto-5′-nucleotidase (CD73)-mediated adenosine generation and signaling in the carotid body (CB). During normoxia/hypoxia, ATP released as a neurotransmitter can be converted to adenosine by the action of ecto-nucleosidetriphosphate diphosphohydrolase (CD39) and CD73. Alternatively, ATP can be converted to adenosine in the type I cell and released via the equilibrative nucleoside transporter (ENT). Adenosine binds to A_2_-receptors on the pre- and post-synaptic membrane to increase baseline activity and overall hypoxic sensitivity. Filled lines denote purinergic signaling, dashed lines denote ion flow.

It is now apparent that adenosine can be generated from extracellular ATP (Figure 1). In the CB, a significant synaptic ATP concentration is due to the tonic vesicular neurosecretion from the type I cell [28] and also through ATP release from type II cells via pannexin-1 channels [29]. There may also be ATP release from red blood cells, although this remains to be clarified in the CB circulation. CD73 (ecto-5′-nucleotidase) catalyzes the formation of adenosine from AMP, following initial breakdown of ATP and adenosine diphosphate (ADP) by CD39 (ecto-nucleosidetriphosphate diphosphohydrolase) [30]. RNA and protein expression of CD73 have now been identified in the CB with the majority being localized to the type I cell [31]. Inhibiting CD73 with α,β-Methylene-ADP (AOPCP) decreases the total pool of extracellular adenosine in the CB [7]. Furthermore, similar pharmacological inhibition of CD73 causes a dramatic reduction in basal chemoafferent activity and blunts the CB response to hypoxia in vitro and the cardiovascular-respiratory response to hypoxia in vivo [22]. Given the high concentrations of AOPCP used in these studies, there is still a need to validate these findings using genetic models or with lower doses of more selective CD73 inhibitors that are becoming available. Consistent with a hypoxia-inducible factor-1 (HIF-1)-dependent elevation of CD73 in other tissues, including hypoxic tumors [32], CB CD73 expression increases in response to chronic hypoxia along with A_2B_-receptors [10,33]. Interestingly, a recent study shows that CD73 expression is elevated in the aged CB, despite an overall reduction in chemoreceptor function [34]. However, a role for CD73 in mediating CB hyperactivity in CIH, SH or HF remains to be elucidated.

### 2.2. Adrenaline

Although adenosine is regarded as the major positive regulator of cAMP in the CB, there are other substances that may elevate cAMP. One of these is adrenaline. Adrenaline binds to β-adrenoceptors, most commonly the β_1_ and β_2_ subtypes which are coupled to the G_αs_ subunit, resulting in cAMP elevation in heart and other tissue [35]. Although there is substantial evidence that adrenaline stimulates breathing [36,37], the direct effect on the CB is less clear. There are reports of exogenous adrenaline administration in vivo causing both increased and decreased CB chemoreceptor discharge [36,38,39]. The effect might be dependent on the dose/concentration used, whilst physiological concentrations selectively act on β-adrenoceptors, supra-physiological levels could act on D_2_-receptors leading to inhibition. This is supported in our own work where we identified a stimulatory effect at 10 nM but an inhibitory action at higher concentrations, as evidenced by a marked reduction in chemoafferent frequency [37,40].

**Figure 2:**
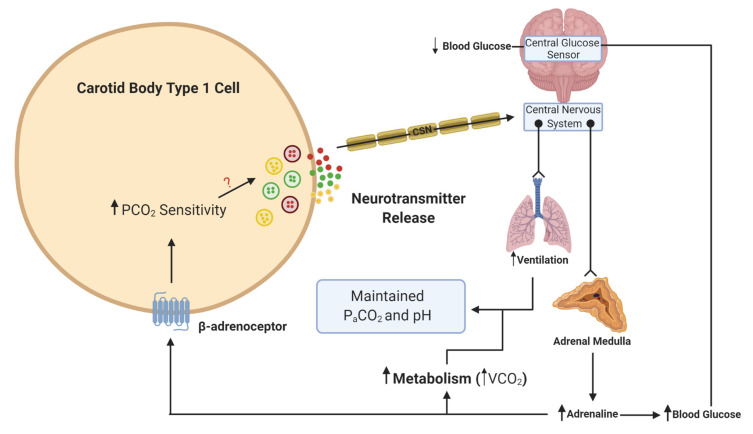
Schematic illustration of adrenaline activation of the carotid body (CB) during hypoglycemia. When blood glucose decreases, this is sensed in the brainstem and leads to a reflex increase in adrenaline release from the adrenal medulla. β-adrenoceptors in type 1 cells will be activated by adrenaline. This activation will lead to an increase in CO_2_ sensitivity, neurotransmitter release and an increase in ventilation. The elevation in ventilation matches the increase in metabolic rate and CO_2_ generation (VCO_2_), such that the overall partial pressure of arterial CO_2_ (P_a_CO_2_) and pH remain constant. The ? denotes that the signaling mechanism linking increased CO_2_ sensitivity with enhanced neurotransmitter release is still unknown. Lines with arrows denote signaling pathways. Lines with circles and chevrons denote efferent neurons.

What is more important to consider is the physiological relevance of endogenous adrenaline. Our own understanding of the effects of endogenous adrenaline have been developed through the study of the counter-regulatory response to hypoglycemia. During hypoglycemia, adrenaline levels rise [41], and hepatic glucose release increases to counter this fall. Multiple studies have now provided evidence of activation of the CB in response to hypoglycemia in animals [42,43] and humans [44,45]. This is essential not only to further augment hepatic glucose release but also to increase ventilation to match the concurrent rise in metabolic rate, initiated by adrenaline [43,46] (Figure 2). In these circumstances, a heightened CB CO_2_ sensitivity allows for increased chemoreceptor discharge and ventilation in the absence of any change in arterial blood gases. Recent evidence has shown that the increase in minute ventilation and CO_2_ sensitivity during hypoglycemia was blunted by propranolol treatment, removal of the adrenal gland and section of the carotid sinus nerve (CSN) [37]. This is consistent with the view that it is adrenaline that activates the CB during hypoglycemia, via β-adrenoceptors. Key experiments are now needed to see if this is also evident in humans. Perhaps surprisingly, no studies have yet looked at a potential role for adrenaline in causing CB hyperactivity in OSA or HF, both of which are associated with a chronic rise in plasma catecholamines. Chronic isoprenaline delivered via osmotic mini-pump does elevate baseline breathing and responses to hypoxia and hypercapnia [40], but as yet, the relevance of this model to pathology remains uncertain and studies in animals and/or humans with HF and CIH are required [47]. Moreover, there is hardly any indication of how adrenaline acts to augment CB chemosensitivity (even the specific receptor subtypes) and there is limited evidence of the impact of beta-blockers on CB function. Key experiments are needed to establish if chronic beta-blocker treatment (which are widely prescribed for heart failure, hypertension and arrhythmia) dampens CB function such that there is increased vulnerability to hypoxic and hypoglycemic exposure. As such, we are just beginning to grasp the importance of adrenaline in mediating CB function in health and disease.

### 2.3. Lactate and Olfr78

Lactate signaling through the GPCR Olfr78 (G_αs_) has gained significant attention based on the finding that Olfr78 mRNA is highly expressed in the murine CB and its global deletion completely abolishes the hypoxic ventilatory response (HVR) [48]. This was coupled with a complete depression of CB chemoafferent activity during hypoxia. Indeed, it is logical that both intracellular and systemic lactate levels increase during hypoxia after partial or complete termination of oxidative phosphorylation in the CB and other tissues, thereby providing a stimulus for Olfr78. The work has now been strengthened by evidence from another laboratory demonstrating a similar depression in the HVR in a different mouse strain [49]. However, this was in spite of severe hypoxia failing to depress the elevation in type I cell [Ca^2+^]_i_ in Olfr78^−/−^, raising questions over the exact location of the Olfr78 signaling within the CB or chemoreflex pathway. Furthermore, this same study observed similar elevations in chemoafferent nerve activity induced by lactate in Olfr78^−/−^ and wildtype (WT) CBs, suggesting that another ligand, and not lactate, activates Olfr78 during hypoxia. In contrast to these studies, it has also been reported that that Olfr78^−/−^ mice maintain a robust HVR and retain type I cell sensitivity to hypoxia [50]. Clearly there is a need to reconcile these findings and in particular to study lactate and Olfr78 in higher species, including humans, which have a larger chemoafferent response to hypoxia and are less susceptible to respiratory alkalosis. In our own studies, we observed that after prolonged exposure to glucose deprivation, which eventually caused chemoafferent excitation, 5 mM lactate acted to suppress rather than excite chemoafferent frequency [51]. Despite being in a different experimental setting, we propose that lactate may have an additional role as an alternative energy source, independent of Olfr78, similar to that seen in the central nervous system (CNS) and peripheral nerves [52,53].

### 2.4. Dopamine and Noradrenaline

The CB contains a large amount of stored catecholamines (CAs) in dense core vesicles, and for its mass, the overall CA content is equivalent only to that seen in adrenal medullary tissue [54]. The enzyme tyrosine hydroxylase (TH), that initiates the synthesis of CAs from tyrosine, has now emerged as a well-established type I cell marker [55]. Dopamine (DA) is the most abundant CA in type I cells, forming more than 50% of the overall CA content [54,56]. There is some evidence of dopamine beta-hydroxylase expression in type I cells, consistent with small amounts of noradrenaline (NA) [57]. Interestingly, dopamine beta-hydroxylase is augmented in CBs of SH rats, possibly indicative of a more pronounced role of NA as a transmitter in pathology [58]. Recently, it has been demonstrated that type I cells contain significant amounts of vesicular monoamine transporter 1 (VMAT1), the enzyme most likely responsible for incorporation of DA and NA into secretory vesicles [59,60]. This process may also be highly sensitive to levels of biotin, a vitamin and coenzyme known to accumulate in type I cells [59]. Future experiments may well evaluate how these enzymes are altered in CB-mediated cardiovascular and metabolic disease.

DA is released from type I cells in abundance during hypoxia [61,62], but its action seems to be autoinhibitory, providing the CB with a degree of inhibitory feedback control via D_2_-receptors [63,64,65,66]. Indeed, it has been suggested that the balance between dopamine (via G_αi_-coupled D_2_-receptors) and adenosine (via G_αs_-coupled A_2B_-receptors) is critical in determining the overall cAMP level and excitability within the type I cell [25]. The increase in [Ca^2+^]_i_ in rat type I cells in response to hypoxia can be attenuated by D_2_-receptor agonists [67]. D_2_-receptors have now also been positively confirmed in the human CB [68] and DA infusion to depress CB function is commonly used to estimate CB contribution to pathophysiological reflexes in humans [69,70,71]. Whether or not DA completely silences the CB chemoafferent discharge in humans is unknown. Exogenous DA does indeed reduce (but not abolish) the HVR in humans, but there is some considerable variability between individuals and not all of the observed effects may be solely attributable to CB inhibition [72,73]. In mice with global deficiency of D_2_-receptors, whilst type I cell neurotransmitter release was enhanced in response to hypoxia, the chemoafferent activity was reduced and the HVR was unchanged [74]. In rats, the HVR can be enhanced rather than depressed, by systemic inhibition of the monoamine oxidase enzyme, which is likely to have increased free DA [75]. These studies are suggestive of additional locations and possible excitatory functions of D_2_-receptors within the CB and/or chemoreflex pathway other than the type I cell in rodents. Any excitatory action does not however seem to involve cAMP sensitive hyperpolarization-activated currents (I_h_), as evidenced by DA causing a reduction in this post-synaptic current through a D_2_-receptor-mediated mechanism [27].

Reverse transcription polymerase chain reaction (RT-PCR) analysis of short- and long-term hypoxic rats’ CBs showed a time-dependent increase in the expression of TH and D_2_-receptor genes [76,77]. After 48 hours of hypoxia, D_2_-receptor mRNA levels decreased, but after 7 days, the expression of D_2_-receptor increased significantly [76]. The alteration in D_2_-receptor expression has been hypothesized to cause a change in DA signaling in the CB which contributes to the changes in ventilatory adaptation observed with long-term hypoxia associated with chronic obstructive pulmonary disease (COPD) or HF [78]. A role for DA in establishing CB hyperactivity in response to CIH has recently been investigated [79]. In this study, CIH augmented CB DA content, CB catecholamine release and arterial blood pressure, but not the HVR. Moreover, the D_2_-receptor antagonist domperidone reversed the elevation in blood pressure and the excessive catecholamine release during hypoxia in CBs isolated from CIH animals. This again goes against the idea that DA is simply an inhibitory neurotransmitter and opens up the possibility that it has an excitatory function in the CB in certain pathological conditions. In this instance, the authors do also point out that some of the effects of domperidone may have been due to changes in either systemic or local CB blood flow.

NA makes up approximately 15–40% of the total catecholamine content of the CB (varying in different species) and is located in the dense core secretory vesicles in type I cells [80,81]. In acute hypoxia, NA is secreted from the type I cells into the extracellular space in proportion to the stimulus intensity [80]. Release of NA from sympathetic terminals and uptake of NA from the circulation also contributes to the total extracellular NA content in the CB. Despite its concentration being significantly lower than that of DA, NA still has an important functional role in CB chemoreception. In a study performed in anaesthetized dogs in the 1970s, it was first observed that infusion of NA into the carotid artery produced a burst of chemoafferent excitation followed by a more sustained inhibition [82]. Similar intracarotid infusions of α_2_-adrenoceptor agonists produced chemoafferent inhibition and blunted the response to hypoxia in anaesthetized cats [83]. Intracarotid injection of NA has been shown to inhibit ventilation in goats, a response that was attenuated by both α-adrenoceptor and D_2_-receptor antagonists [84]. Sectioning of sympathetic nerves innervating the CB in cats was shown to have no effect on baseline chemoafferent activity but did augment the frequency during hypoxia [85]. Direct application of NA to the whole ex vivo CB or dissociated type I cells blunts neurotransmitter release, Ca^2+^ currents and the rise in cAMP during hypoxia, effects which are again dependent on α_2_-adrenoceptor activation [86,87]. Thus, there is a strong body of evidence to suggest that endogenous NA released from sympathetic nerves and type I cells promotes inhibitory feedback via G_αi_-coupled α_2_-adrenoceptors. In contrast, we have seen that venous infusion of NA in anaesthetized rats elevates ventilation that is partially blocked by section of the CSN [40]. It is possible that this method of administration produced a more sustained/intense vasoconstriction to arterioles supplying the CB, leading to a significant reduction in blood flow and local hypoxia. Alternatively, wide-spread delivery of NA could promote the release of intermediate substances into the systemic circulation that act indirectly on the CB to evoke chemostimulation. Clearly, there is a need to unify these contradictory findings. The impact of sympathetic stimulation on CB blood flow requires more investigation and especially in conditions where there is a known increase in sympathetic tone in other vascular beds. Furthermore, it is currently unknown what the chronic effect of elevated plasma NA could be on CB blood flow and function, especially common in conditions such as OSA, SH, HF and pheochromocytoma.

## 3. G_αq_-Protein-Coupled Receptor Signaling in the Carotid Body

### 3.1. Angiotensin II

Angiotensin II (Ang II) is a powerful hormone that influences many organs and its level is increased in important cardiovascular diseases including HF, hypertension and chronic kidney disease. A wealth of evidence has also shown that Ang II acutely increases CB chemoafferent frequency with a threshold in the pico/nano-molar range [88,89]. Rises in [Ca^2+^]_i_ initiated by Ang II can be attenuated by losartan, an angiotensin 1 (AT_1_)-receptor antagonist [90]. RT-PCR analysis revealed that both AT_1a_ and AT_1b_ subtypes are expressed in type I cells and immunohistochemistry has confirmed the presence of AT_1_-receptor protein [90,91]. Combined with the finding that an AT_2_-receptor antagonist was not able to prevent the Ang II-induced increase in [Ca^2+^]_i_, these results suggest that AT_1_-receptors are responsible for modifying CB activity via Ang II. AT_1_-receptors are coupled to the G_αq_ protein and its activation leads to the generation of IP_3_, via phospholipase C, causing the release of Ca^2+^ from intracellular stores [92]. It is now thought that this rise in Ca^2+^ is sufficient to activate the non-selective cation channel which contributes to cellular depolarization [93]. Repetitive application of Ang II can also promote sensory long-term facilitation (sLTF) of chemoafferent nerve activity, an effect reliant on activation of nicotinamide adenine dinucleotide phosphate (NADPH) oxidase and ROS generation [89]. AT_1_-receptor signaling is also key to causing CB sensory long-term facilitation (sLTF) and persistent sympathoexcitation following acute intermittent hypoxia, in a protein kinase C (PKC), ROS-dependent manner [94,95]. Whether or not the response to prolonged Ang II desensitizes due to receptor phosphorylation, β-arrestin recruitment and internalization is yet to be studied in the CB. Recent reports have indicated that AT_1_-receptor inhibition does not reduce the HVR or chemoreflex in healthy humans before or after a short period (8 h) of hypoxia [96]. Furthermore, raising serum Ang II in healthy individuals does not lead to chemoreflex sensitization [97]. This possibly indicates that Ang II and AT_1_-receptor signaling might be more important in pathology or short/long-term CB adaptation rather than setting chemosensitivity in healthy individuals.

Chronic hypoxia has been shown to increase AT_1a_ and AT_1b_-receptor mRNA expression in type I cells as well as CSN sensitivity to Ang II [98]. In situ hybridization, PCR and Western blot analysis have confirmed the presence of components of the renin-angiotensin system (RAS), including angiotensinogen and angiotensin-converting enzyme (ACE), in type I cells of the CB, indicative of a local system [99]. These components are elevated in rat CBs exposed to chronic hypoxia and CIH [99,100]. AT_1_-receptor protein is also increased in CBs following CIH [101]. It has therefore been suggested that Ang II may play a role in causing CB hyperactivity in diseases associated with chronic hypoxia and/or CIH, e.g., in OSA. To support this, heightened sympathetic activity following exposure to CIH is prevented in rats treated with losartan, an AT_1_-receptor inhibitor [101]. Translational findings in humans are currently limited. However, in a recent clinical trial, it was observed that losartan reduced systolic and diastolic blood pressure in OSA patients without modifying muscle-sympathetic outflow or ventilation during hypoxia [102]. OSA was fairly well established in these patients and as such, there may have been irreversible epigenetic remodeling of CB function [103], accounting for the apparent lack of impact of losartan on CB function. Future studies could evaluate if losartan or similar agents are more effective in protecting against the development of hypertension in newly diagnosed or more-mild OSA patients.

In animal models of HF, augmented CB Ang II signaling is also suggested to increase basal and hypoxic sympathetic outflow and contribute to neurogenic hypertension [104]. AT_1_-receptor antagonists reduce chemoafferent firing frequency and renal sympathetic nerve activity in rabbits with HF but not controls [104]. Furthermore, The AT_1_-receptor antagonist, L-158,809 (1 μM), significantly decreases the sensitivity of voltage-dependent K^+^ current to hypoxia in type I cells isolated from CH but not control rabbits [105]. These increased actions of Ang II in HF are reported to be mediated by increased NADPH-oxidase activity and ROS generation [106]. Clinical trials are now called for to directly assess the role of Ang II and Ang II antagonists in mediating CB hyperactivity and hypertension in human patients with HF.

### 3.2. Serotonin (5-HT)

Serotonin (5-HT) is another excitatory neurotransmitter in the CB and the enzymes involved in its biosynthesis (tryptophan hydroxylase) and transport (5-HT transporters) have been identified in type I cells, indicating that 5-HT is synthesized locally [107,108]. During normoxia, the release of 5-HT occurs spontaneously from type I cells in large clusters (ca 20 cells) to establish baseline spike-like depolarizations [109,110]. Exogenous 5-HT evokes a significant increase in [Ca^2+^]_i_ in a distinct population of type I cells [111]. 5-HT binds to the G_αq_-linked 5-HT_2A_-receptor subtype expressed in type I cells and the stimulatory effect is via a PKC-mediated inhibition of the Ca^2+^-activated K^+^ current [110]. Hypoxia induces depolarization of type I cells leading to neurotransmitter release, including 5-HT. 5-HT_2_-receptor antagonists reduce both the number and magnitude of type I cell spike depolarizations and the hypoxia-stimulated rise in [Ca^2+^]_i_ [112]. However, nerve-recording studies have demonstrated that 5-HT does not significantly alter the peak response to hypoxia, but does prolong the duration of the response [113]. 5-HT has also been implicated in contributing to chemoafferent hyperactivity in the CB during pathophysiological conditions associated with CIH [114]. 5-HT release and 5-HT_2_-receptor activation are crucial for NADPH oxidase (NOX) activation during CIH [114]. Hypoxia induces a significant increase in 5-HT release from type I cells in CIH-treated animals, which in turn leads to an increase in PKC activity and NADPH-oxidase 2-derived ROS generation, which is suggested to cause persistent chemoafferent hyperactivity [115]. Taken together, these results suggest a role for 5-HT in setting CB excitability and in both normal and pathological conditions. However, as with Ang II, it is not known whether the response to prolonged 5-HT desensitizes and if some of the longer-lasting effects of 5-HT in pathology are dependent on β-arrestins. In addition, selective targeting of the CB with 5-HT_2_-receptor antagonists is yet to be trialed in humans with OSA. 

## 4. Receptors with Dual G_αs_/G_αq_ Signaling

### 4.1. Pituitary Adenylate Cyclase-Activating Polypeptide (PACAP) and the PAC_1_-Receptor

Pituitary adenylate cyclase-activating polypeptide (PACAP) is a neuropeptide that was originally identified in the hypothalamus and was observed to be capable of increasing cAMP content in pituitary cell cultures [116]. However, since PACAP can be detected in peripheral blood [117], there is also the potential for systemic actions, including on the CB. PACAP was initially suggested to exert an excitatory influence on the type I cell by inhibiting background TASK currents and raising [Ca^2+^]_i_ [118]. This is now thought to occur via activation of pituitary adenylate cyclase-activating polypeptide type 1 (PAC_1_)-receptors. Interestingly, the activation of PAC_1_-receptors can lead to stimulation of both G_αs_ and G_αq_ signaling pathways. In experiments utilizing the ex vivo intact CB preparation, it was shown that prolonged PACAP application (50 min, 200 nM) caused a biphasic response characterized by an initial short-lasting peak 2–3-fold elevation in chemoafferent discharge, followed by a drop and plateau at approximately 1.5–2-fold above baseline [119]. Whilst inhibitors of PKA and EPAC did inhibit chemoafferent responses, these were modest compared to the effect of the PLC inhibitor U73122, which strongly suppressed both the peak and steady-state responses [119]. Thus, although there are roles for both G_αs_ and G_αq_, it is more likely that in this case, G_αq_ predominates.

Of particular importance is the potential link between PACAP and sudden infant death syndrome (SIDS) in African Americans [120]. In this pilot study, a significant association was observed between SIDS and a single nucleotide polymorphism in exon 2 of the PACAP gene, but only in African American infants and not Caucasians [120]. The reason for the observed differences is still to be elucidated. Further studies are required to explore this relationship in much larger patient cohorts and in different ethnicities. However, it is known that neonatal animals that lack the PACAP gene have high mortality due to significantly reduced respiratory responses to both hypoxia and hypercapnia, and a high incidence of prolonged apneas [121]. Furthermore, neonatal mice lacking the PAC_1_-receptor have a reduced HVR compared to WT littermates [122]. Administration of PACAP-38, an exogenous form of PACAP, in the rat in situ working heart-brainstem preparation induced a significant increase in respiratory frequency, an effect that was abolished by CB denervation [123]. PACAP-38 also stabilized breathing in this preparation, however, this was not modified by CB denervation, indicative of a central, CB-independent action. Thus, the role for PACAP and the CB in SIDS is still to be defined, and there is an urgent need to identify more personalized protective therapies for vulnerable infants at particularly high risk.

### 4.2. Endothelin

**Figure 3:**
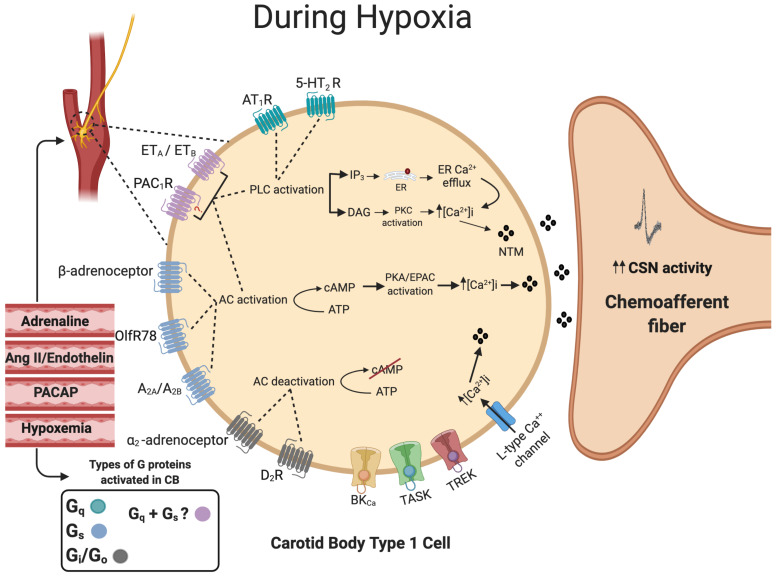
Summary of G-protein signaling in the carotid body type I cell. The activation of G_q_-protein-coupled receptors (Green) activates phospholipase C (PLC), which in turn leads to the formation of both inositol trisphosphate (IP_3_) and diacylglycerol (DAG). IP_3_ will bind to the endoplasmic reticulum (ER), causing ER Ca^2+^ efflux. DAG will activate protein kinase C (PKC). The activation of G_s_-protein-coupled receptors (Blue) will activate transmembrane adenylyl cyclase (tmAC), leading to an increase in intracellular cyclic adenosine monophosphate (cAMP) production, which will activate protein kinase A (PKA). The activation of pituitary adenylate cyclase-activating polypeptide type 1 (PAC_1_) and endothelin (ET) receptors (Pink) could activate both G_q_ and G_s_ mechanisms. Stimulation of G_i_-protein-coupled receptors (Gray) predominantly by dopamine will inhibit tmAC activity, which in turn decreases cAMP. The overall balance between the concentration of external ligands and the extent of activation of each of the G_s_, G_i_ and G_q_ pathways is capable of acutely fine-tuning the type I cell hypoxic sensitivity. Many of these receptors and signaling pathways are also involved in physiological and pathological carotid body adaptation. The dashed circle identifies the position of the CB and type I cells at the carotid bifurcation. Dashed lines denote receptor activation linking to specific downstream enzymes. Filled lines denote further downstream or upstream signaling cascades.

Endothelin (ET) is a 21 amino acid peptide most commonly known for its ability to cause potent vasoconstriction. The G-protein-coupled receptors ET_A_ and ET_B_ are expressed in the CB type 1 cells as well as in the surrounding blood vessels [124]. ET_A_- and ET_B_-receptors can be coupled to multiple G-proteins, including G_αq_, G_αs_ and G_αi,_. Given that ET causes type I cell stimulation and an increase in [Ca^2+^]_i_, they are most likely to be linked with G_αq_ and G_αs_ in this tissue [125]. The main function of ET signaling appears to be in mediating CB adaptation to chronic hypoxia and CIH in adults [126,127] and neonates [128]. In adult rats, chronic hypoxia upregulates ET_A_- and ET_B_-receptor expression in the type I cell and ET_A_-selective antagonists have a strong impact on chemoafferent discharge, producing ca 11% inhibition before chronic hypoxia, increasing to ca 50% inhibition after 16 days of chronic hypoxia [129,130]. In chronic hypoxia, increased endothelin signaling seems to overlap with enhanced generation of nitric oxide (NO), a substance capable of both increasing and decreasing CB chemosensitivity dependent on the specific microdomain in which it is produced [129,131]. Further work is necessary to define the exact role and location of this increased NO generation in chronic hypoxia. In contrast, exposure to CIH appears to increase expression of ET_B_ but not ET_A_ [124]. Non-selective ET-receptor antagonists have a greater inhibitory effect on chemoafferent activity in CIH compared to control rat and cat CBs [132,133]. In a recent study, it was suggested that the augmented ET signaling includes upregulation of PLC and PKC [134]. In neonatal rat CBs, the elevation in chemoafferent activity is dependent on ET_A_-receptor stimulation and ROS generation [128]. These important findings can be advanced further by evaluating ET signaling and CB hyperactivity in humans and in other CB-mediated pathology, such as HF and spontaneous/essential hypertension. A summary of G-protein signaling in the CB type I cell is presented in Figure 3.

## 5. Other G-Protein Signaling Mechanisms

In addition to being a principal excitatory neurotransmitter, it has been proposed that ATP has an autocrine and paracrine function in the CB to provide a negative feedback mechanism and amplify the effects of adenosine, respectively [135,136]. ATP receptors that are also GPCRs include the P2Y_1_- and P2Y_2_-receptor subtypes which are coupled to the G_αq_ protein and have been identified in the CB. P2Y_2_-receptors are localized to the glia-like sustentacular type II cells and administration of ATP leads to an increase in [Ca^2+^]_i_ and the opening of pannexin-1 channels, which allow the release of ATP [135,137]. This ATP-induced ATP release allows for further breakdown of ATP via CD73 to form adenosine, which can then act on A_2A_- and A_2B_-receptors, enhancing adenosine chemoexcitation in the CB, suggesting cross-communication between type I and type II cells [6]. However, ATP can also act on P2Y_1_-receptors located on the type I cell, providing a negative feedback mechanism at high extracellular concentrations. Studies have shown that high extracellular levels of ATP had no effect on the resting [Ca^2+^]_i_ but were able to restrict the increase in [Ca^2+^]_i_ during hypoxia [136]. Although the exact mechanism is unknown, activation of the P2Y_1_-receptor has been suggested to close background ion channels other than the common TASK-like K^+^ or Na^+^ channels, resulting in type I cell hyperpolarization [136].

Similarly, as well as being a key excitatory neurotransmitter, acetylcholine (ACh) also has a modulatory function acting through G_αq_-coupled muscarinic receptors [138,139]. Interestingly, stimulation of type I cells by methylcholine can be inhibited by histamine, H_3_-receptor (G_αi_) agonists and SQ22536, suggestive of interaction between G_αi_ and G_αq_ pathways [140,141]. The precise point(s) at which multiple different G-protein pathways converge remains to be revealed. Exploring the cross-talk between multiple different GPCRs and downstream signaling pathways could be an interesting area for investigation in CB-mediated cardiovascular disease [78].

## 6. Conclusions

G-proteins play a crucial role in CB function both in normal physiology and pathology. Activation of GPCRs in the CB can cause either stimulation or the inhibition of chemoafferent activity dependent on intricate balance between numerous different neuromodulators, GPCRs and second messenger systems. Ultimately, changes in the output from the CB mediated by GPCRs will have important systemic effects, including variations in ventilation, heart rate, blood vessel vasoconstriction and blood glucose. Importantly, GPCRs have a very significant part in evoking CB hyperactivity, hypertension and cardiac arrhythmia associated with key conditions such as OSA and HF. Despite this, there is still no therapy used clinically that directly targets the CB. Thus, translational studies are urgently required to evaluate if drugs targeting GPCRs can effectively protect against or reverse CB-mediated cardiovascular disease in humans.

## Abbreviations

5-HT	Serotonin
8-SPT	8-(p-Sulfophenyl)theophylline
AC	Adenylyl cyclase
tmAC	Transmembrane adenylyl cyclase
ACE	Angiotensin-converting enzyme
ACh	Acetylcholine
ADP	Adenosine diphosphate
cAMP	Cyclic adenosine monophosphate
Ang II	Angiotensin II
AT_1_	Angiotensin 1 receptor
AOPCP	α,β-Methylene-ADP
CAs	Catecholamines
CB	Carotid body
CD39	Ecto-nucleosidetriphosphate diphosphohydrolase
CD73	Ecto-5′-nucleotidase
CH	Chronic hypoxia
CIH	Chronic intermittent hypoxia
COPD	Chronic obstructive pulmonary disease
CSN	Carotid sinus nerve
DA	Dopamine
DAG	Diacylglycerol
ENT	Equilibrative nucleoside transporter
EPACs	Exchange proteins activated by cAMP
ER	Endoplasmic reticulum
ET	Endothelin
GPCR	G-protein-coupled receptor
HF	Heart failure
HIF-1	Hypoxia-inducible factor-1
HVR	Hypoxic ventilatory response
IP_3_	Inositol trisphosphate
NA	Noradrenaline
NADPH	nicotinamide adenine dinucleotide phosphate
NOX	NADPH oxidase
OSA	Obstructive sleep apnea
PAC_1_	pituitary adenylate cyclase-activating polypeptide type 1
PACAP	Pituitary adenylate cyclase-activating polypeptide
PKA	Protein kinase A
PKC	Protein kinase C
PLC	Phospholipase C
RAS	Renin-angiotensin system
Rf	Respiratory frequency
ROS	Reactive oxygen species
SH	Spontaneous/essential hypertension
SIDS	Sudden infant death syndrome
sLTF	Sensory long-term facilitation
TASK	Twik-related acid-sensitive K^+^ channel
TH	Tyrosine hydroxylase
VCO_2_	CO_2_ production
VMAT1	Vesicular monoamine transporter 1
WT	Wildtype
